# Neighborhood Socioeconomic Disadvantage and Childhood Body Mass Index Trajectories From Birth to 7 Years of Age

**DOI:** 10.1097/EDE.0000000000001420

**Published:** 2021-10-18

**Authors:** Samuli Rautava, Olli Turta, Jussi Vahtera, Jaana Pentti, Mika Kivimäki, Jamie Pearce, Ichiro Kawachi, Päivi Rautava, Hanna Lagström

**Affiliations:** From the a Department of Pediatrics, University of Turku and Turku University Hospital, Turku, Finland; b Department of Pediatrics, University of Helsinki and New Children’s Hospital, Helsinki University Hospital, Helsinki, Finland; c Department of Public Health, University of Turku, Turku, Finland; d Centre for Population Health Research, University of Turku and Turku University Hospital, Turku, Finland; e Department of Epidemiology and Public Health, University College London, London, United Kingdom; f Clinicum, Faculty of Medicine and Helsinki Institute of Life Science, University of Helsinki, Helsinki, Finland; g Centre for Research on Environment, Society & Health, School of GeoSciences, University of Edinburgh, Edinburgh, United Kingdom; h Department of Social and Behavioral Sciences, Harvard T. H. Chan School of Public Health, Boston, MA; i Turku University Hospital, Research Services, Turku, Finland.

**Keywords:** Children, Neighborhood socioeconomic disadvantage, Overweight, Obesity, Body mass index

## Abstract

**Background:**

The epidemic of increasing childhood overweight and obesity is a major global health concern, with local contextual factors identified as possible contributors. Robust research is needed to establish an evidence base supporting health policy decisions to reverse the trend. We aimed to examine the association between neighborhood socioeconomic disadvantage and trajectories of body mass index (BMI) from birth to age 7.

**Methods:**

The present study included 11,023 children born within the Southwest Finland Birth Cohort who were free of severe conditions affecting growth with adequate exposure and growth data. We obtained child growth data until school age from municipal follow-up clinics. We based cumulative childhood neighborhood socioeconomic disadvantage on the average annual income, unemployment, and level of education in a residential area defined using a geographic grid at a spatial resolution of 250 m by 250 m.

**Results:**

Cumulative neighborhood socioeconomic disadvantage was associated with distinct childhood BMI z score trajectories from birth to age 7. Despite being born in the lowest BMI z scores, children growing up in disadvantaged neighborhoods subsequently exhibited a trajectory of increasing BMI z scores starting at 4 years of age, ending up with a higher risk of overweight at the end of the follow-up (30%) as compared with children living in more affluent neighborhoods (22%). The corresponding risk of obesity was 5 % for those in affluent neighborhoods and 9 % and those in disadvantaged neighborhoods.

**Conclusion:**

Cumulative exposure to neighborhood socioeconomic disadvantage is independently associated with unfavorable BMI development and obesity in childhood.

## Abstract 2

Supplemental Digital Content is available in the text.

With the prevalence of overweight and obesity increasing worldwide, the obesity pandemic constitutes a major public health concern.^1^ Overweight is rapidly becoming more prevalent amongst children, and children who are overweight tend to manifest with obesity as adults^2^ and exhibit increased blood pressure and unfavorable lipid profiles later in life.^3^ Identifying children at high risk of developing obesity and the developmental stage at which they enter different trajectories of weight gain is critical for optimal targeting of interventions aiming to reduce the obesity pandemic.

Factors related to adverse socioeconomic circumstances, such as low family socioeconomic status (SES), maternal obesity, high birth weight, and short or absent breastfeeding may increase childhood obesity risk.^4–6^ The association between neighborhood socioeconomic disadvantage, overweight, and obesity is also well established, but mostly in studies of adults.^7^ It has been suggested that neighborhood disadvantage may expose a child to increased risk of obesity directly and indirectly via maternal habits and behavior,^8–13^ which in turn may be affected by various aspects of the built environment including access to healthy or unhealthy food retailing, neighborhood walkability, and the availability of green spaces.^14^ Existing evidence shows that childhood neighborhood socioeconomic disadvantage is associated with increased risk of obesity in school age,^15^ adolescence,^16^ and early adulthood.^13,17^ However, the specific age at which the effect of neighborhood socioeconomic disadvantage on child overweight and obesity risk might become manifest is not known.

We hypothesized that cumulative neighborhood socioeconomic disadvantage is associated with unfavorable childhood body mass index (BMI) trajectories from birth to school age. To test this hypothesis, we sought to establish the association between neighborhood socioeconomic disadvantage and childhood BMI development from birth to 7 years of age in a population-based longitudinal birth cohort linked to detailed residential histories. The prospective register-based data and annual measurements of height and weight enabled us to assess the BMI trajectories at different exposure levels although controlling for maternal risk factors and parental socioeconomic status (SES). Our results will aid in targeting preventive measures to specific neighborhoods and age groups, which is likely to improve the efficacy of the interventions.

## METHODS

### Study Population

**FIGURE 1.**
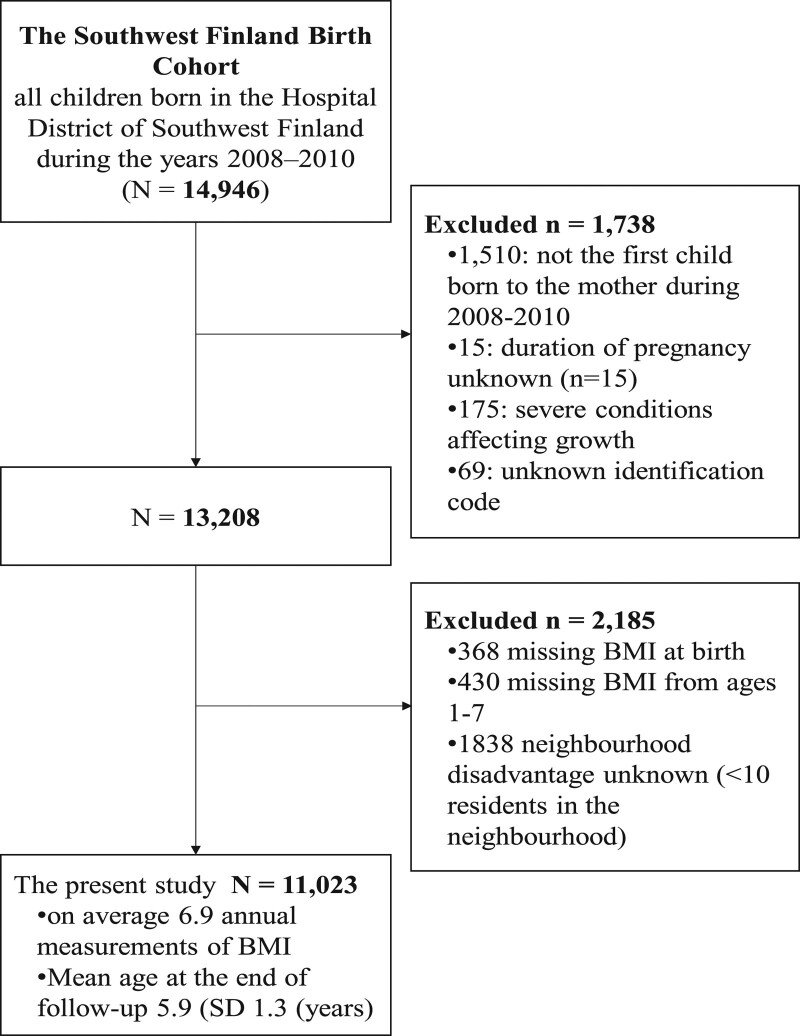
Flowchart summarizing the subjects included in the study from the Southwest Finland Birth Cohort. The severe conditions affecting growth that served as criteria for exclusion included genetic syndromes, substantial congenital heart disease, malignancies, and endocrine and growth disturbances requiring growth hormone therapy.

This study is based on the Southwest Finland Birth Cohort, which consists of all 14,946 children born in the Hospital District of Southwest Finland during the years 2008–2010.^18^ This hospital district included two hospitals, and there were no other private or municipal maternity hospitals in the area at the time of the study. Consequently, the study cohort consists of all children born in the geographical area during the 3-year period. In this study, the first child born during this time period from each mother was included, excluding those with chronic conditions affecting growth, missing information on height or weight at birth, no growth measurements between ages 1 and 7 or missing information on neighborhood socioeconomic disadvantage, leaving 11,023 children in the analytic sample (Figure 1). All data regarding the children in the cohort were collected from municipal and national registers. The study was approved by the Ethics Committee of the Finnish Institute for Health and Welfare. The legal basis for processing of personal data is public interest and scientific research (EU General Data Protection Regulation 2016/679 (GDPR), Article 6(1)(e), and Article 9(2)(j); Data Protection Act, Sections 4 and 6).

### Pre- and Perinatal Characteristics

Pre- and perinatal characteristics including child birthweight and length, sex, preterm birth (birth occurring before 37 weeks of gestation), maternal age, mode of delivery (vaginal or cesarean section), primiparity (no previous deliveries), single parenthood (not married or cohabiting at the time of childbirth), smoking during pregnancy (yes or no), maternal weight and height before pregnancy, gestational diabetes mellitus, other medical conditions the mother manifested with during pregnancy [mental and behavioral disorders (ICD-10 codes F00-F99), diseases of the circulatory (I00-I99), respiratory (J00-J99), digestive (K00-K93) or genitourinary (N00-N99) systems], and parental SES were extracted from the national register on parturients, deliveries, and births maintained by the Finnish Institute for Health and Welfare. We identified gestational diabetes using ICD-10 code O24. Obesity was defined as prepregnancy BMI > 30 kg/m^2^. Parental SES, based on mother’s self-reported occupation, was classified as higher-grade nonmanual, lower-grade nonmanual, manual, student, full-time mother, or other. We obtained information on the primary language of the mother from the Population Register Center. Mothers were classified as having immigrant background if their primary language was not Finnish or Swedish (the official languages spoken in Finland).

### Child Weight and Length Development During the First 7 Years of Life

We obtained child growth data until school age from municipal follow-up clinics. According to Finnish legislation, all municipalities are under the obligation to organize a minimum of 15 preventive childcare visits during the first 6 years of the child’s life. Children enter the school health care system in the autumn term of the year they turn 7 years of age and, consequently, the municipal well-baby clinic follow-up is completed at the age of 6–7 years. The follow-up clinics use standardized methods for the measurement of length/height and weight provided by the Finnish Institute for Health and Welfare. The anthropometric data at birth and closest to the time points of 1 and 2 years of age (within 3 months), and 3, 4, 5, 6, and 7 years of age (within 6 months) were used in the analyses. The World Health Organization growth charts^19^ were used to obtain age-specific z scores for BMI. We used the BMI z scores +1 SD to estimate the prevalence of overweight and +2 SD to estimate obesity. The numbers of participants with available height and weight measurement data at each time point are presented in eTable 1; http://links.lww.com/EDE/B854.

### Characteristics of Local Environments

Data regarding neighborhood social disadvantage were derived from a grid database established and maintained by Statistics Finland. The database contains socioeconomic information from each residence at a spatial resolution of 250 m by 250 m.^20^ The grid data were obtained with 5-year intervals between 1990 (the first time point available from Statistics Finland) and 2015. The neighborhood disadvantage score is based on the proportion of adults with low education, the unemployment rate, and the average annual income of households in each 250 m × 250 m grid area.^21,22^ We replaced missing data (i.e., areas with fewer than 10 residents in the neighborhood) with the mean neighborhood disadvantage score of the eight adjacent map squares. For each of the three variables, we derived a standardized z score based on the total Finnish population (mean = 0, SD = 1). We then calculated a score for neighborhood disadvantage by taking the mean value across the three z scores. Higher scores on the continuous index denote greater disadvantage. For the statistical analyses, the neighborhood disadvantage score was classified into four categories based on national means as follows: <–1 SD (lowest disadvantage), –1 to 0 SD, ≥0 to 1 SD, and >1 SD (highest disadvantage).

We obtained high-quality residential mobility data, based on a complete history of the residential addresses with latitude and longitude coordinates, from the Population Register Center for each mother and her child until the child was 7 years old. Using open-source Geographical Information Systems (QGIS, http://www.qgis.org/en/site/), data on the cumulative residential neighborhood disadvantage for each time point were linked to the cohort participants’ home addresses by the latitude and longitude coordinates. We calculated time-dependent socioeconomic disadvantage score weighted by residential time at each location for each study subject.

### Statistical Analysis

Missing information for binary confounders (immigrant background n = 6, single parenthood n = 18, smoking n = 23, and maternal obesity n = 48) was imputed using the mode value. With the repeated measured outcome and exposure missing data in between birth and the last measurement (3.6% and 1.5% of all 76,334 observations, respectively) were imputed using the mean of observed values of the person. To examine the associations of the pre- and perinatal characteristics (potential confounders) with the neighborhood disadvantage categories at birth, we used the chi square test for categorical variables and general linear model for continuous variables. The same models were used to examine the associations of potential confounders with BMI Z score at birth and at the last measurement.

To model the trajectories of childhood BMI until school age, we used marginal structural models with generalized estimating equations (GEE) and inverse probability weighting. This approach allows adjustment for confounding and selection bias owing to measured time-varying covariates affected by prior exposure and outcome.^23,24^ For weighted estimation of the parameters in the marginal structural models we fitted three models: the exposure model, the censoring model, and the structural (i.e., weighted) model. Two weights for each observation were estimated, one to adjust for exposure selection bias and the other to adjust for dropout from the follow-up. We calculated the weights using predicted values obtained from logistic regression of the probability of being censored between T_i_ and T_i+1_, according to exposure and covariates at T_i_, and at T_i+1_, respectively (for the Directed Acyclic Graph, see eFigure 1; http://links.lww.com/EDE/B854). Similarly, the weights for exposure selection were calculated using multinomial logistic regression. Stabilized weights for the final models were calculated by multiplying the inverse probability weights for exposure selection with those for censoring. Distribution of the stabilized weight by age is shown in eFigure 2; http://links.lww.com/EDE/B854.

At birth, the participants lived in 3,791 different neighborhoods (mean population density 86). Only 13% of the neighborhoods had more than five cohort members. There was only one cohort member in 51% of the neighborhoods. Altogether 6,546 (59%) participants moved to other neighborhoods during the follow-up. Consequently, there was no clustering by neighborhood to be corrected in the models.

With the marginal structural models including the age-disadvantage interaction term, we estimated the mean level of BMI z score and the 95% confidence intervals (CI) at each age by the categories of cumulative neighborhood disadvantage from birth onward. Sex differences in the BMI trajectories were tested in a model including the interaction term “sex*age* cumulative neighborhood disadvantage.” As there was no interaction (*P* = 0.54), the results are shown for boys and girls combined. The fully adjusted model controlled for child sex and preterm birth, maternal risk factors, and parental SES. Using contrast, we calculated the mean difference between categories of cumulative neighborhood disadvantage at each age using the lowest category of disadvantage as a reference group. We replicated these analyses using the continuous disadvantage score as the measure of exposure at each age. We also estimated the changes in BMI z score within each category of neighborhood disadvantage level in three different age periods: from birth to age 1 year, from 1 to 4 years, and from 4 to 7 years.

As a sensitivity analysis, we calculated the observed trajectories of BMI z score according to the individual components of the neighborhood disadvantage score: educational level, unemployment rate and average household income in the neighborhood. We also performed additional analyses using the alternative cutoffs of 0.5 SD and 1.5 SD to examine whether the findings were sensitive to specific cutoffs.

Finally, we examined the risk of overweight or obesity at 6–7 years of age by the level of cumulative neighborhood disadvantage. For these Poisson regression analyses, we included all children who completed the follow-up, adjusting the models for child sex and preterm birth, maternal risk factors, and parental SES. The results are expressed as risk across the categories of neighborhood disadvantage and the risk ratios and their 95% CIs compared with the lowest disadvantage category. Sex differences in the associations were tested in a model including the interaction term “sex*cumulative neighborhood disadvantage.” As there were no interactions (test for overweight *P* = 0.43 and for obesity *P* = 0.43), the results are shown for boys and girls combined. We performed all analyses using the SAS software version 9.4 (SAS Institute Inc., Cary, NC).

## RESULTS

The clinical characteristics of the study cohort are presented in Table 1. Altogether 1,246 (11%) of the children were born in neighborhoods with highest socioeconomic disadvantage whereas 1,412 (13%) of the children were born in the most affluent neighborhoods. Mothers whose children were born in neighborhoods with highest disadvantage were younger, and more often single parents at the time of delivery, from immigrant background, smokers, and manifested with obesity as compared with those whose children were born in the most affluent neighborhoods (Table 1).

**TABLE 1.** Pre- and Perinatal Characteristics of the Participants and Their Association With Neighborhood Socioeconomic Disadvantage at Birth

		Neighborhood Disadvantage^a^			
		<–1 SD (Lowest) N (%)	–1 to 0 SD N (%)	>0 to 1 SD N (%)	>1 SD (Highest) N (%)
	11,023	1,412 (13)	5,163 (47)	3,202 (29)	1,246 (11)
Maternal characteristics					
Age (years), mean (SD)	30.0 (5.1)	32.0 (4.3)	30.8 (4.8)	28.8 (5.3)	27.5 (5.6)
Primiparous, N (%)					
No	5,523 (50)	874 (62)	2,570 (50)	1,456 (46)	623 (50)
Yes	5,500 (50)	538 (38)	2,593 (50)	1,746 (55)	623 (50)
Mode of delivery, N (%)					
Vaginal	9,539 (86)	1,223 (87)	4,473 (87)	2,752 (86)	1,091 (88)
Cesarean section	1,484 (14)	189 (13)	690 (13)	450 (14)	155 (12)
Single parenthood at birth, N (%)					
No	10,352 (94)	1,393 (99)	4,974 (96)	2,913 (91)	1,072 (86)
Yes	671 (6)	19 (1)	189 (4)	289 (9)	174 (14)
Immigrant, N (%)					
No	10,054 (91)	1,382 (98)	4,940 (96)	2,864 (89)	868 (70)
Yes	969 (9)	30 (2)	223 (4)	338 (11)	378 (30)
Smoking during pregnancy, N (%)					
No	9,762 (89)	1,361 (96)	4,733 (92)	2,710 (85)	958 (77)
Yes	1,261 (11)	51 (4)	430 (8)	492 (15)	288 (23)
Obesity before pregnancy,^b^ N (%)					
No	9,773 (89)	1,289 (91)	4,607 (89)	2,806 (88)	1,071 (86)
Yes	1,228 (11)	123 (9)	556 (11)	396 (12)	175 (14)
Gestational diabetes mellitus, N (%)					
No	9,332 (85)	1,226 (87)	4,359 (84)	2,700 (84)	1,047 (84)
Yes	1,691 (15)	186 (13)	804 (16)	502 (16)	199 (16)
Other medical conditions,^c^ N (%)					
No	10,690 (97)	1,373 (97)	5,019 (97)	3,106 (97)	1,192 (96)
Yes	333 (3)	39 (3)	144 (3)	96 (3)	54 (4)
Parental socioeconomic status, N (%)					
Higher-grade nonmanual	2,297 (21)	458 (32)	1,265 (25)	476 (15)	98 (8)
Lower-grade nonmanual	2,203 (20)	385 (27)	1,150 (22)	548 (17)	120 (10)
Manual	3,234 (29)	300 (21)	1,418 (28)	1,082 (34)	434 (35)
Student	1,206 (11)	63 (5)	504 (10)	443 (14)	196 (16)
Full-time mother	465 (4)	25 (2)	132 (3)	161 (5)	147 (12)
Other	1,618 (15)	181 (13)	694 (13)	492 (15)	251 (20)
Child characteristics					
Sex of the child, N (%)					
Boy	5,635 (51)	739 (52)	2,631 (51)	1,657 (52)	608 (49)
Girl	5,388 (49)	673 (48)	2,532 (49)	1,545 (48)	638 (51)
Preterm birth, N (%)					
No	10,566 (96)	1,362 (96)	4,940 (96)	3,076 (96)	1,188 (95)
Yes	457 (4)	50 (4)	223 (4)	126 (3)	58 (5)
Duration of pregnancy (weeks), mean (SD)	39.9 (1.5)	39.8 (1.5)	39.9 (1.5)	39.9 (1.5)	39.8 (1.6)
Birth weight (g), mean (SD)	3,527 (506)	3,571 (495)	3,536 (507)	3,510 (507)	3,482 (511)

^a^Standardized z score based on the total Finnish population.

^b^BMI > 30.

^c^Other medical conditions the mother manifested with during pregnancy are mental and behavioral disorders, diseases of the circulatory, respiratory, digestive, or genitourinary systems.

The associations between risk factors and children’s BMI z score at birth and in the last measurement during follow-up are presented in Table 2. Young maternal age, primiparity, single parenthood, immigrant background, smoking during pregnancy, and medical conditions during pregnancy were associated with a lower BMI z score at birth. At the end of the follow-up, single parenthood and smoking during pregnancy were associated with a higher BMI z score although preterm birth showed no association. Prepregnancy obesity, GDM, and low-parental SES were associated with a higher BMI z score at both time points.

**TABLE 2.** Associations Between pre- and Perinatal Characteristics of the Participants and Mean (95% CI) BMI z Score at Birth and at the End of Follow-up (Mean 5.9 Years)

	At Birth			At the End of Follow-up		
	Mean	95% CI		Mean	95%CI	
Maternal age at birth (years)						
15–29	0.16	0.13	0.19	0.39	0.36	0.42
30–49	0.19	0.17	0.22	0.37	0.35	0.40
Primiparous						
No	0.37	0.34	0.40	0.41	0.38	0.44
Yes	–0.02	–0.04	0.01	0.36	0.33	0.39
Mode of delivery						
Vaginal	0.20	0.18	0.22	0.38	0.36	0.40
Cesarean section	0.05	–0.01	0.10	0.42	0.37	0.48
Single parenthood at birth						
No	0.18	0.17	0.20	0.37	0.35	0.40
Yes	0.05	–0.03	0.13	0.52	0.44	0.60
Immigrant						
No	0.19	0.17	0.21	0.39	0.36	0.41
Yes	0.01	–0.05	0.08	0.36	0.29	0.43
Smoking during pregnancy						
No	0.19	0.17	0.21	0.35	0.33	0.37
Yes	0.05	–0.01	0.10	0.66	0.60	0.72
Obesity before pregnancy^a^						
No	0.15	0.13	0.17	0.31	0.29	0.33
Yes	0.37	0.32	0.43	0.97	0.92	1.03
Gestational diabetes mellitus						
No	0.16	0.14	0.18	0.35	0.33	0.37
Yes	0.28	0.24	0.33	0.57	0.52	0.62
Other medical conditions						
No	0.18	0.16	0.20	0.38	0.36	0.40
Yes	–0.05	–0.16	0.07	0.38	0.26	0.49
Parental socioeconomic status						
Higher-grade nonmanual	0.15	0.11	0.20	0.26	0.22	0.31
Lower-grade nonmanual	0.21	0.17	0.25	0.37	0.32	0.41
Manual	0.22	0.18	0.25	0.51	0.47	0.55
Student	0.15	0.09	0.21	0.30	0.24	0.36
Full-time mother	0.26	0.17	0.35	0.50	0.40	0.60
Other	0.08	0.03	0.13	0.35	0.30	0.40
Sex of the child						
Boy	0.12	0.10	0.15	0.38	0.35	0.41
Girl	0.23	0.20	0.26	0.39	0.36	0.41
Preterm birth						
No	0.25	0.23	0.27	0.39	0.37	0.41
Yes	0.16	0.13	0.19	0.33	0.24	0.43

^a^BMI > 30.

^b^Other medical conditions the mother manifested with during pregnancy are mental and behavioral disorders, diseases of the circulatory, respiratory, digestive, or genitourinary systems.

Predictors for exposure selection and censoring are shown in eTable 2; http://links.lww.com/EDE/B854. Previous exposure to neighborhood disadvantage was the strongest predictor of current exposure. Previous BMI was not associated with current exposure. Both previous exposure and previous BMI predicted censoring the OR for dropout per 1 SD higher neighborhood disadvantage being 0.92 (95% CI = 0.86, 0.98) and the corresponding OR per 1 SD higher in BMI being 0.95 (95% CI = 0.92, 0.98).

**FIGURE 2.**
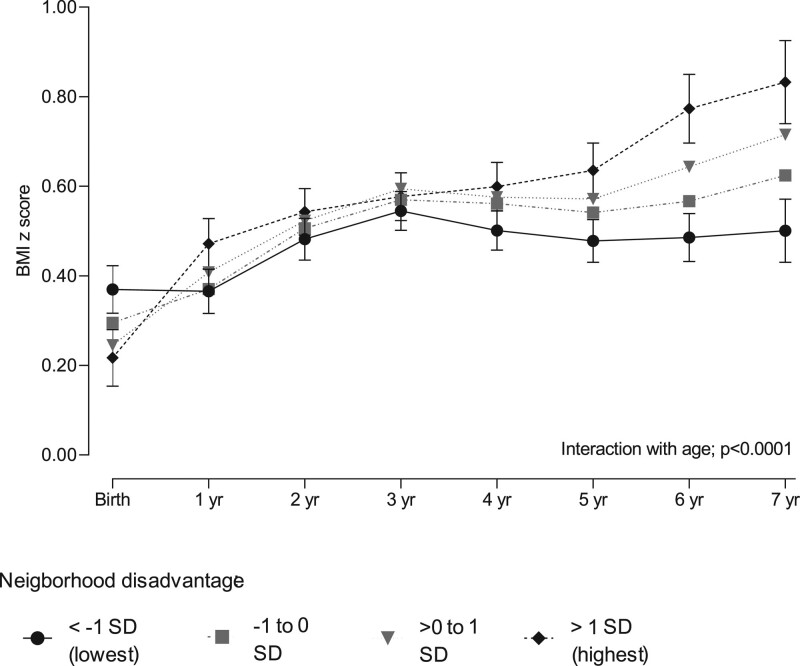
BMI z score trajectories in children exposed to cumulative neighborhood socioeconomic disadvantage. BMI z scores are expressed as mean values and their 95% confidence intervals from birth to 7 years of age. The marginal structural GEE models with inverse probability weighting are adjusted for child sex, preterm birth, maternal age, primiparity, single parenthood, immigrant background, smoking during pregnancy, prepregnancy obesity, gestational diabetes mellitus, other maternal medical conditions during pregnancy, and parental socioeconomic status. We detected no interaction with child sex (*P* = 0.54). Disadvantage categories are based on national standardized mean score.

Figure 2 shows the BMI trajectories estimated by the marginal structural models with inverse probability weighting adjusted for confounders and eFigure 3; http://links.lww.com/EDE/B854 shows the observed associations of neighborhood disadvantage and its components from birth until the age of 7 years. The pattern for the overall level of disadvantage was replicated for each individual component of disadvantage including educational level, unemployment rate, and average household income in the neighborhood.

Childhood neighborhood socioeconomic disadvantage was associated with distinct temporal BMI trajectories during the mean follow-up of 5.9 years (test of interaction with time; adjusted *P* < 0.0001), with no difference between the sexes (test of interaction; *P* = 0.54). The mean BMI z score at birth was inversely associated with the level of neighborhood socioeconomic disadvantage. In the adjusted analysis, the lowest mean BMI z score at birth (0.22; 95% CI = 0.15, 0.28) was observed in children with highest neighborhood socioeconomic disadvantage, whereas the highest mean BMI z score (0.37; 95% CI = 0.32, 0.42) was found in children in the most affluent neighborhoods (mean difference –0.15; 95% CI = –0.24, –0.07) (eTable 3; http://links.lww.com/EDE/B854). From birth to the age of 1 year, the increase in BMI z score in children living in the most disadvantaged neighborhoods was 0.26 (95% CI = 0.18, 0.33), while in the children living in the most affluent neighborhoods no change was observed (0.00; 95% CI = –0.07, 0.06) (eTable 4; http://links.lww.com/EDE/B854). Between the ages of 2 and 4 years, the difference in BMI changes between the exposure groups diminished. However, the BMI z score trajectories began to diverge between the ages 4 to 7 with an increase in children living in the most disadvantaged neighborhoods 0.23 (95% CI = 0.16, 0.31) and no change in children living in the most affluent areas (0.00; 95% CI = –0.06, 0.06). At the age of 7 years, the adjusted mean BMI z score was 0.83 (95% CI = 0.74, 0.93) for those living in the most disadvantaged neighborhoods and 0.50 (95% CI = 0.43, 0.57) for those living in the most affluent neighborhoods; mean difference 0.33 (95% CI = 0.22, 0.45). In a sensitivity analysis using a continuous neighborhood disadvantage score, the trend per 1 SD increase in disadvantage was negative at birth –0.04 (95% CI = –0.06, –0.01) and 0.12 (95% CI = 0.08, 0.16) at age 7 (eTable 3; http://links.lww.com/EDE/B854). Sensitivity analyses using alternative cutoff points for the categories of neighborhood disadvantage indicated that the findings remained essentially the same with different cutoffs (eFigure 4; http://links.lww.com/EDE/B854).

**FIGURE 3.**
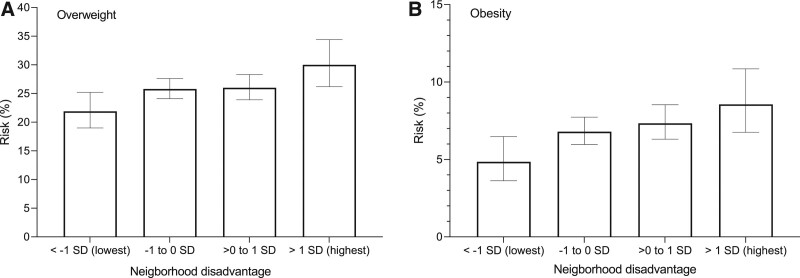
Cumulative neighborhood disadvantage and risk of (A) overweight and (B) obesity at age 6–7 years. Overweight is defined as BMI z score greater than +1 SD and obesity as BMI z score greater than +2 SD. Only those with a completed follow-up were included in the analysis (N = 8,021). The models were adjusted for child sex, preterm birth, maternal age, primiparity, single parenthood, immigrant background, smoking during pregnancy, prepregnancy obesity, gestational diabetes mellitus, other medical conditions during pregnancy, and parental socioeconomic status. The whiskers represent the 95% confidence interval. We detected no interaction with child sex for overweight (*P* = 0.43) or obesity (*P* = 0.43).

Neighborhood socioeconomic disadvantage was linked to increasing risk of overweight and obesity by school age in children with complete follow-up (Figure 3, Table 3). The adjusted risk of overweight was 30 % (95% CI = 26.2, 34.4) in the children exposed to highest neighborhood disadvantage and 22 % (95% CI = 19.0, 25.2) in children living in the most affluent areas indicating a 1.37-fold (95% CI = 1.12, 1.67) risk. The corresponding risks for obesity were 9% (95% CI = 6.8, 10.9) and 5 % (95% CI = 3.6, 6.5), risk ratio 1.77 (95% CI = 1.21, 2.57). There was no difference between the sexes (test of interaction; *P* > 0.40).

**TABLE 3.** Cumulative Neighborhood Disadvantage and Risk of Overweight and Obesity in Children Aged of 6–7 Years

Disadvantage^a^	Risk (%)	95% CI		RR	95% CI	
Outcome overweight						
< –1 SD (lowest)	21.9	19.0	25.2	1.00 (ref)		
–1 to 0 SD	25.8	24.1	27.6	1.18	1.01	1.37
>0 to 1 SD	26.0	23.9	28.3	1.19	1.01	1.40
>1 SD (highest)	30.0	26.2	34.4	1.37	1.12	1.67
Outcome obesity						
< –1 SD (lowest)	4.8	3.6	6.5	1.00 (ref)		
–1 to 0 SD	6.8	6.0	7.7	1.40	1.03	1.90
>0 to 1 SD	7.3	6.3	8.5	1.51	1.10	2.09
>1 SD (highest)	8.6	6.8	10.9	1.77	1.21	2.57

^a^Standardised z score based on the total Finnish population.

Risk Ratios (RR) and their 95% confidence intervals (CI) are adjusted for child sex, preterm birth, mother’s age, primiparousness, marital status, immigration, smoking during pregnancy, prepregnancy obesity, gestational diabetes mellitus, other medical conditions during pregnancy, and parental socioeconomic status.

Interaction with sex for overweight *P* = 0.43 and for obesity *P* = 0.43.

## DISCUSSION

We found cumulative neighborhood socioeconomic disadvantage to be associated with childhood BMI trajectories from birth to school age in a large, population-based, prospective birth cohort with serial anthropometric measurements, and statistical analyses adjusting for a large number of potential confounding factors. After being born with the lowest BMI z scores, the children growing up in the most disadvantaged neighborhoods exhibited an increase in BMI z scores during the first year of life. This was not seen in children from the most affluent neighborhoods. After a modest increase in BMI z scores between the ages of 1 and 4 years in children from both disadvantaged and advantaged neighborhoods, a trajectory of increasing BMI z scores was observed in children exposed to highest cumulative neighborhood disadvantage from age 4 years to age 7. In contrast, no change in BMI z scores was seen in the children least exposed to neighborhood disadvantage. These findings interestingly correspond to data published from the United States, according to which African–American children exhibited lower birth weight compared with White children but experienced steeper BMI trajectories later in childhood.^25^ Children with low birthweight often exhibit rapid postnatal growth, which is reportedly associated with increased risk of obesity.^6^ In the present study, however, the difference in BMI trajectories between children who lived in disadvantaged and advantaged neighborhoods was not explained by preterm birth, primiparity, single parenthood, immigrant background, smoking during pregnancy, prepregnancy obesity, gestational diabetes mellitus, or other medical conditions during pregnancy. Furthermore, the accelerated increase in BMI z scores leading to overweight and obesity began as late as age 4. Thus, rather than being driven by prenatal influences, our results are consistent with the hypothesis that exposure to neighborhood socioeconomic disadvantage constitutes an important risk factor for the development of childhood obesity.

The association of neighborhood socioeconomic status with health has previously mostly been investigated in adults. Among the middle-aged population, cumulative neighborhood socioeconomic disadvantage has been associated with increased cardiometabolic risk factors as well as increased incidence of diabetes mellitus and major cardiovascular diseases.^17,26–29^ A number of studies have found an association between childhood neighborhood disadvantage and BMI or the risk of overweight or obesity in school age,^15^ adolescence,^16^ and early adulthood.^13^ Our current data demonstrate a similar association in Finland, a country with small socioeconomic differences and inequalities.

Our study has several strengths which increase the reliability of the results. The prospective study design in an unselected population-based cohort of all children born in the Southwest Finland between 2008 and 2010 support the validity of our results, but further research is needed to examine whether our findings are generalizable across different settings and countries. The classification of neighborhoods by socioeconomic disadvantage was based on objective measures of household income, unemployment rate, and level of education with high geographical resolution. The quality of the residential mobility data in Finland is high as all residential addresses are accurately recorded in the national population register. We were able to accurately calculate cumulative exposure to neighborhood socioeconomic disadvantage based on the residential history of the children using geographically precise and regularly updated spatial information. The prospective study design with serial standardized anthropometric measurements provides reliable growth data and allows assessing the age at which the BMI trajectories diverge. The marginal structural models with inverse probability weighting correct for the differences in the baseline characteristics between included and censored participants and minimize the potential of selection bias that could be introduced because of these differences.^23^ Moreover, the findings remained essentially the same with different cutoffs for neighborhood disadvantage categories. In the main analysis, we used predefined cutoffs based on the total Finnish population to facilitate comparisons with other studies on the Finnish population.

Previous studies on the associations of neighborhood socioeconomic status with childhood obesity have variably taken into consideration individual-level factors, which are known to affect childhood obesity development. In the present study, single parenthood at the time of childbirth, smoking during pregnancy, prepregnancy obesity, and parental SES were all associated with both childhood neighborhood socioeconomic disadvantage and BMI z score at the end of the follow-up. In the statistical model adjusted for these confounding factors, the association between cumulative neighborhood disadvantage and childhood BMI z score development remained evident.

This study has a number of limitations. Data on paternal education or income were not available. It is therefore possible that some of the results are owing to the influence of individual SES. However, the strong association between parental SES and neighborhood disadvantage at birth implies that a major bias owing to such residual confounding is unlikely. Furthermore, data on paternal BMI or smoking had not been recorded. We did not have data on the age at onset of puberty, which has been associated with both individual SES^30,31^ and the risk of obesity.^32,33^ However, it is unlikely that differences in puberty onset explain the observed associations given that the follow-up ended at the age of 7 years while the onset of puberty typically occurs considerably later in children with and without obesity.^32,33^

A relatively large number of subjects were lost to follow-up during the study period. Adherence to the visits at municipal clinics is generally high, and the proportion of those not attending any visits has been estimated to be as low as 0.5% based on vaccination coverage.^34,35^ A small proportion of the missing data may be explained by the study subjects moving to geographical areas outside of Southwest Finland. It is therefore likely that the reason for the missing anthropometric data is mostly related to gaps in data acquisition from municipalities using different electronic record systems.

Observational studies have suggested that neighborhood socioeconomic disadvantage has wide-ranging health effects, such as increased risk of obesity in school age,^15^ adolescence,^16^ and early adulthood^13,17^ and diabetes in adulthood.^36^ The evidence is often obtained from direct comparisons of disease incidence between different residential neighborhoods and is therefore subject to health-related selection into residential environments. This can introduce a self-selection bias if health-related issues affect people’s choices of moving to a particular area. Previous studies, however, suggest that selection bias is unlikely to explain the association between neighborhood disadvantage and health-related outcomes. In contrast, experimental evidence supports the hypothesis that neighborhood characteristics directly affect health. For example, in the Moving to Opportunity residential mobility experiment, adults living in disadvantaged areas in five US cities were randomly assigned the opportunity to move to a less disadvantaged area.^26^ Follow-up 10 to 15 years later showed that people who moved to less-disadvantaged areas had a lower prevalence of obesity and diabetes than members of the control group who were not offered the same opportunity. Natural experiments and analyses using individuals as their own controls have reported similar findings on changes in neighborhood characteristics and health-related outcomes^21,22,28^

In the present study, we were able to control for self-selection in several ways. First, we had access to comprehensive residential mobility data from birth until age 7, which allowed us to control for the effects of moving between residential areas. Second, we were able to control for a wide range of confounders potentially affecting selection of place of residence, such as single parenthood, immigrant background, smoking, obesity, maternal medical conditions during pregnancy and parental SES. In our data, those lost to follow-up were more likely to live in affluent areas and had a lower BMI z score than the stayers, but these predictors of censoring were controlled for in the MSM analysis. Thus, selective retention is an unlikely source of major bias in this study.

The proximal cause of obesity is excessive energy intake related to expenditure, which is usually explained by a combination of an energy-rich diet and a sedentary lifestyle. The distal causes underlying the development of obesity are more difficult to discern. Clustering of several risk factors, such as maternal prepregnancy obesity, smoking during pregnancy, single parenthood, immigrant background, gestational diabetes mellitus, and other medical conditions during pregnancy and low-parental SES likely explains part of the association between high neighborhood socioeconomic disadvantage and childhood BMI. However, children exposed to high neighborhood disadvantage exhibited unfavorable BMI development even after adjusting for these factors. Local environment and neighborhood socioeconomic status have previously been reported to be associated with maternal breastfeeding behavior,^8,9^ which may in turn modulate the risk of childhood obesity.^37^ Neighborhood characteristics including access to physical activity facilities, playgrounds, and parks; the proximity of food retail establishments; and walkability and perceived neighborhood safety have also been associated with childhood obesity risk in some but not all studies^38–42^ and may mediate the associations observed in the present study.

Public planning and funding of neighborhood development plays a major role in all these aspects of the local environment. We found that children growing up in the most disadvantaged neighborhoods exhibited increasing BMI z scores particularly after 4 years of age and a high prevalence of obesity at 7 years of age. These results provide new insight into the intergenerational link between neighborhood disadvantage and the risk of childhood obesity and, if corroborated by future studies, may be of benefit to health policy makers.

## Supplementary Material


