# Supplementary material for: Neighborhood Socioeconomic Disadvantage and Childhood Body Mass Index Trajectories From Birth to 7 Years of Age
Source: Epidemiology. 2021 Oct 18;33(1):121–30. doi: 10.1097/EDE.0000000000001420 (PMC8614531; doi:10.1097/EDE.0000000000001420)
Supplement: Supplementary file 1 [file ede-33-121-s001.docx]

**Supplementary Appendix**

**Supplement to: Rautava S. et al. Neighborhood socioeconomic disadvantage and childhood body mass index trajectories from birth to 7 years of age**

**eTable 1**. Number of participants with available height and weight measurements at each age.

| Age (yrs) | Total | Boys | Girls |
| --- | --- | --- | --- |
| Birth | 11023 | 5635 | 5388 |
| 1 | 11023 | 5635 | 5388 |
| 2 | 10879 | 5569 | 5310 |
| 3 | 10628 | 5435 | 5193 |
| 4 | 10360 | 5310 | 5050 |
| 5 | 9685 | 4950 | 4735 |
| 6 | 8021 | 4102 | 3919 |
| 7 | 4715 | 2423 | 2292 |

**eTable 2.** Predictors for exposure selection and censoring

| **Exposure weight denominator model** | | | |
| --- | --- | --- | --- |
| **Effect** | **DF** |  | **Pr > ChiSq** |
|  |  | **Chi-Square** |  |
| Age | 3 | 8.9 | 0.03 |
| Sex | 3 | 5.3 | 0.15 |
| Preterm birth | 3 | 2.3 | 0.50 |
| Immigrant background | 3 | 90.6 | <0.001 |
| Primiparity | 3 | 19.2 | <0.001 |
| Mode of delivery | 3 | 2.4 | 0.49 |
| Single parenthood at birth | 3 | 49.9 | <0.001 |
| Smoking during pregnancy | 3 | 71.9 | <0.001 |
| Maternal age | 3 | 40.1 | <0.001 |
| Obesity before pregnancy† | 3 | 4.8 | 0.19 |
| Gestational diabetes mellitus | 3 | 7.1 | 0.07 |
| Other medical conditions§ | 3 | 1.0 | 0.81 |
| Parental socioeconomic status | 15 | 79.7 | <0.001 |
| Neighbourhood disadvantage at birth^a^ | 9 | 213.5 | <0.001 |
| BMI z-score at birth | 3 | 1.1 | 0.77 |
| Previous neighbourhood disadvantage^a^ | 9 | 11348.6 | <0.001 |
| Previous BMI z-score | 3 | 2.2 | 0.53 |
|  |  |  |  |
| **Censoring weight denominator model** | | | |
| **Effect** | **DF** | **Chi-Square** | **Pr > ChiSq** |
| Age | 1 | 3346.1 | <0.001 |
| Sex | 1 | 0.2 | 0.65 |
| Preterm birth | 1 | 0.1 | 0.71 |
| Immigrant background | 1 | 4.4 | 0.04 |
| Primiparity | 1 | 17.3 | <0.001 |
| Mode of delivery | 1 | 0.1 | 0.73 |
| Single parenthood at birth | 1 | 0.4 | 0.53 |
| Smoking during pregnancy | 1 | 0.0 | 0.86 |
| Maternal age | 1 | 0.3 | 0.59 |
| Obesity before pregnancy^b^ | 1 | 7.1 | 0.01 |
| Gestational diabetes mellitus | 1 | 2.3 | 0.13 |
| Other medical conditions^c^ | 1 | 1.9 | 0.17 |
| Parental socioeconomic status | 5 | 16.9 | 0.005 |
| Neighbourhood disadvantage at birth^a^ | 3 | 3.1 | 0.37 |
| BMI z-score at birth | 1 | 1.0 | 0.31 |
| Last neighbourhood disadvantage^a^ | 3 | 10.4 | 0.02 |
| Last BMI z-score | 1 | 11.8 | <0.001 |

^a^ standardized z score based on the total Finnish population

^b^ BMI>30

^c^ other medical conditions the mother manifested with during pregnancy are mental and behavioural disorders, diseases of the circulatory, respiratory, digestive or genitourinary systems

**eTable 3**. Association of cumulative neighbourhood disadvantage with BMI at each age group. Models adjusted for child sex, preterm birth, mother's age, primiparity, marital status, immigrant background, smoking during pregnancy, pre-pregnancy obesity, gestational diabetes mellitus, other medical conditions during pregnancy and parental SES.

|  | **Continuous disadvantage**^a^ | | |  | **Categorical disadvantage**^b^ | | | | | | | | | | |  |
| --- | --- | --- | --- | --- | --- | --- | --- | --- | --- | --- | --- | --- | --- | --- | --- | --- |
|  |  | | |  | **-1 to 0 vs <-1** | | |  | **0 to 1 vs <-1** | | |  | **>1vs<-1** | | | |
| **Age** | **Trend/ 1SD** | **95% CL** | |  | **Mean diff** | **95% CL** | |  | **Mean diff** | **95% CL** | |  | **Mean diff** | **95% CL** | | |
| Birth | -0.04 | -0.06 | -0.01 |  | -0.07 | -0.13 | -0.02 |  | -0.12 | -0.19 | -0.06 |  | -0.15 | -0.24 | -0.07 | |
| 1 y | 0.05 | 0.03 | 0.08 |  | 0.01 | -0.05 | 0.06 |  | 0.04 | -0.02 | 0.10 |  | 0.11 | 0.03 | 0.18 | |
| 2 y | 0.04 | 0.01 | 0.06 |  | 0.02 | -0.02 | 0.07 |  | 0.04 | -0.01 | 0.10 |  | 0.06 | -0.01 | 0.13 | |
| 3 y | 0.03 | 0.01 | 0.05 |  | 0.03 | -0.02 | 0.07 |  | 0.05 | 0.00 | 0.10 |  | 0.03 | -0.04 | 0.10 | |
| 4 y | 0.04 | 0.02 | 0.07 |  | 0.06 | 0.02 | 0.10 |  | 0.07 | 0.02 | 0.12 |  | 0.10 | 0.03 | 0.17 | |
| 5 y | 0.06 | 0.04 | 0.09 |  | 0.06 | 0.02 | 0.11 |  | 0.09 | 0.04 | 0.15 |  | 0.16 | 0.08 | 0.23 | |
| 6 y | 0.11 | 0.07 | 0.14 |  | 0.08 | 0.03 | 0.14 |  | 0.16 | 0.09 | 0.22 |  | 0.29 | 0.20 | 0.38 | |
| 7 y | 0.12 | 0.08 | 0.16 |  | 0.12 | 0.05 | 0.20 |  | 0.22 | 0.13 | 0.30 |  | 0.33 | 0.22 | 0.45 | |

^a^ National standardised mean score

^b^ Cutpoints -1, 0 and +1 SD. Reference <-1SD (lowest disadvantage)

**eTable 4**. Change in BMI score at different age periods by level of neighbourhood disadvantage.

Models adjusted for child sex, preterm birth, mother's age, primiparity, marital status, immigrant background, smoking during pregnancy, pre-pregnancy obesity, gestational diabetes mellitus, other medical conditions during pregnancy and parental SES.

|  | **Period** | | | | | | | | | | | |
| --- | --- | --- | --- | --- | --- | --- | --- | --- | --- | --- | --- | --- |
|  | **From birth to 1 year** | | |  | **From 1 to 4 year** | | |  | **From 4 to 7 year** | | |  |
| **Disadvantage^a^** | **Mean diff** | **95% CL** | |  | **Mean diff** | **95% CL** | |  | **Mean diff** | **95% CL** | |  |
| < -1 SD  (lowest) | -0.004 | -0.065 | 0.057 |  | 0.136 | 0.094 | 0.178 |  | -0.001 | -0.057 | 0.056 |  |
| -1 to 0 SD | 0.075 | 0.042 | 0.108 |  | 0.191 | 0.168 | 0.214 |  | 0.063 | 0.035 | 0.090 |  |
| >0 to 1 SD | 0.163 | 0.121 | 0.205 |  | 0.168 | 0.137 | 0.198 |  | 0.140 | 0.100 | 0.179 |  |
| >1 SD  (highest) | 0.255 | 0.184 | 0.326 |  | 0.127 | 0.072 | 0.183 |  | 0.233 | 0.158 | 0.309 |  |

^a^National standardized mean score

**eFigure 1.** DAG showing the time-dependent relationships between the exposure and outcome variables.

Maternal pre- and perinatal characteristics were age, primiparity, mode of delivery, single parenthood, immigration, smoking, obesity, diabetes and diseases during pregnancy.

Child characteristics were sex and full-term vs pre-term birth.

.
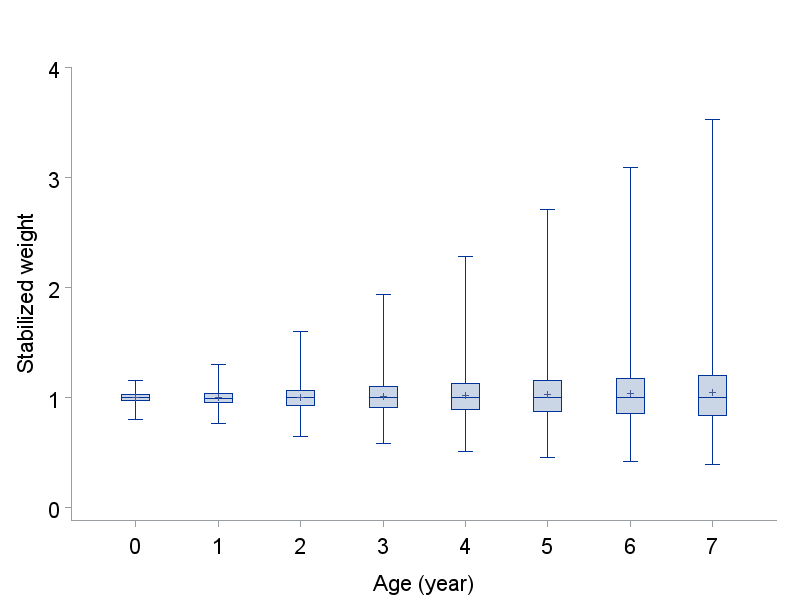


**eFigure 2.** Distribution of stabilised weight by age.

Box and whiskers: min, 1^st^ quartile, median, 3^rd^ quartile, max; plus: mean

**eFigure 3. Observed mean BMI Z-score by components of neighbourhood disadvantage and the overall cumulative disadvantage from birth to 7 year**

The socioeconomic composition for each grid comprised the average annual income of households, the mean number of years of education of residents over 18 years of age, and the proportion of unemployed in adult residents belonging to the labour force. The cut-off is based on national standardised mean score

eFigure 4. Estimated mean BMI Z-score from birth to age 7 years by cumulative disadvantage with alternative cut-off points.

The marginal structural GEE models with inverse probability weighting are adjusted for child sex, preterm birth, maternal age, primiparity, single parenthood, immigrant background, smoking during pregnancy, pre-pregnancy obesity, gestational diabetes mellitus, other medical conditions during pregnancy and parental socioeconomic status.

The cut-off is based on national standardised mean score.
